# Antioxidant Properties of Pyroligneous Acid Obtained by Thermochemical Conversion of *Schisandra chinensis* Baill

**DOI:** 10.3390/molecules191220821

**Published:** 2014-12-12

**Authors:** Chunhui Ma, Wei Li, Yuangang Zu, Lei Yang, Jian Li

**Affiliations:** 1 State Engineering Laboratory for Bioresource Eco-Utilization, Northeast Forestry University, Harbin 150040, China; E-Mails: mchmchmchmch@163.com (C.M.); zygorl@163.com (Y.Z.); 2 College of Material Science and Engineering, Northeast Forestry University, Harbin 150040, China; E-Mail: liwei19820927@126.com

**Keywords:** *Schisandra chinensis* Baill, pyrolysis, pyroligneous acid, antioxidant activity, GC-MS

## Abstract

Sustainable development of renewable resources is a major challenge globally. Biomass is an important renewable energy source and an alternative to fossil fuels. Pyrolysis of biomass is a promising method for simultaneous production of biochar, bio-oil, pyroligneous acid (PA), and gaseous fuels. The purpose of this study was to investigate the pyrolysis process and products yields of *Schisandra chinensis* fruits with different pyrolysis powers. The obtained PA was extracted with organic solvents, including ethyl formate, dichloromethane, methanol and tetrahydrofuran. The antioxidant activities, including the free radical scavenging activity and ferric reducing power, of the PA extracts were investigated. The synthetic antioxidants butylated hydroxyanisole and butylated hydroxytoluene were used as positive controls. A dichloromethane extract of PA showed excellent antioxidant properties compared to the other extracts. The chemical compositions of the PA extracts were determined by GC-MS, and further proved that the dichloromethane extract had the best antioxidant characteristics among the extracts tested.

## 1. Introduction

Biomass is an important renewable resource, and its use for energy production provides economic security that is less environmentally damaging than that of other energy sources. Biomass can be converted by thermochemical processes, including pyrolysis, combustion, gasification, and liquefaction. Pyrolysis is an efficient method for utilization of biomass, especially for agricultural countries with large quantities of available biomass by-products [1,2]. The products of biomass pyrolysis include char, gas, tar and pyroligneous acid (PA).

Also known as wood vinegar or pyroligneous liquor, PA is a complex mixture of water, alcohols, organic acids, phenolics, aldehydes, ketones, esters, furan and pyran derivatives, hydrocarbons, and nitrogen compounds [3,4]. The composition and yield of PA depends on the species the biomass was derived from, and pyrolysis conditions. PA is a reddish-brown, acidic, water-soluble wood distillate, which is used in pesticides, refined food additives [5], and smoke flavoring [6]. PA is useful for soil improvement, and especially for the control of fungal and termite infestations [7,8]. PA also exhibits excellent antioxidant activity. PA from bamboo has superoxide anion scavenging activity and antioxidant activity [9]. Antioxidant and free radical scavenging activities have been reported for PAs from *Rhizophora apiculata* [10], walnut shells [11], and hickory shells [12]. PA has potential as a natural antioxidant because it is rich in phenolic compounds and can be used as a food antioxidant [11].

Antioxidants have become important because of their role in health and their effects on cardiovascular disease, atherosclerosis, cancer, and aging. Many antioxidant compounds from plant sources have been identified as free radical scavengers [13,14]. In recent years, the search for naturally occurring antioxidants for use in food and medicine has intensified in order to replace synthetic antioxidants [15,16], which are being restricted because of their side effects (e.g., carcinogenicity) [17]. A number of synthetic antioxidants, such as 2-*tert*-butyl-4-methoxyphenol and 3-*tert*-butyl-4-methoxyphenol (butylated hydroxyanisole, BHA), and *tert*-butylhydroquinone are added to foodstuffs, but their use has come under scrutiny because of toxicity issues [18]. Therefore, attention has been directed towards the discovery of natural antioxidants from plant sources. Crude extracts of plant materials rich in polyphenols are increasingly of interest to the food industry because of their capacity to retard oxidative degradation of lipids and thereby improve the quality and nutritional value of food [13].

The composition and yield of PA depends on the pyrolysis process conditions and the biomass raw materials. Extensive studies of the chemical compositions and applications of PAs from oak, sakura, green tea, bamboo, eucalyptus, mangrove, rosemary and waste biomass have been conducted [7,10,19]. However, the themochemical conversion of *Schisandra chinensis* Baill. (*S. chinensis*) and chemical composition and antioxidant activity of *S. chinensis* PA have not been studied in detail.

Dried fruit of *S. chinensis* are important in herbal medicines and as a food additive in China [20]. They are used extensively in Korea and Japan as a tonic, sedative and astringent agent to treat various diseases [21,22,23]. Modern pharmacological research has shown that *S. chinensis* has antioxidant, antitumor [24], anti-hepatotoxic, detoxificant, anticarcinogenic [25], and anti-inflammatory [26] activity, and can act on the central nervous system. In addition, it can reduce fatigue and increase endurance, which can contribute to improved physical performance in sports [27]. In China, it is also used as a flavor agent and food additive when stewing fish and meat and making soup, tea, yogurt and porridge [28,29,30,31]. The chemical composition of *S. chinensis* fruits includes essential oil terpenoids [32], polysaccharides [33], anthocyanins [34], organic acids, vitamins, tannins [35], and biphenyl cyclooctene lignans [36] was shown in Table 1.

**Table 1:** Chemical composition of *S. chinensis* fruits.

Chemical Composition	Content (%)	Reference
Essential oil	1.2–3.0	[32]
Polysaccharides	1.2–2.2	[33]
Anthocyanins	2.0–3.2	[34]
Terpenoids	<1.5	[35]
Organic acids (citric, malic, fumaric and tartaric acid)	<1.0	[35]
Vitamins C and E	<0.5	[35]
Tannins	<1.5	[35]
Biphenyl cyclooctene lignans and derivatives	7.2–19.2	[36]

This study aimed to investigate the pyrolysis of *S. chinensis*, which can be used to obtained the natural antioxidant *S. chinensis* PA and to reduce environmental pollution from fossil fuels. Another objective of this study was to evaluate the phenolic content, radical scavenging activity, and reducing power of *S. chinensis* PA extracts in ethyl formate, dichloromethane, methanol, and tetrahydrofuran. The extracts were obtained after pyrolysis under different heating conditions. The results could be used for development of antioxidants and preservatives from *S. chinensis* PA.

## 2. Results and Discussion

### 2.1. Influence of Moisture Content and Molding Temperature on the Briquetting Effect

Dried residues with different moisture contents (3.5%, 4.0%, 5.0%, 5.5%, 6.5%, 8.0%, 9.5%, 10.5%, 11.5%, 13.0%, 14.0%, 15.5%, 16.0%, 17.5%) were put into the hopper, respectively, and then the electric heating tube was opened, the molding temperature was kept at 200 °C, and the extrusion machine was started, driving the spiral propellers to push the dried residues into the molding sleeve. The briquetting effect of molding rods is shown in Table 2. When the residual moisture was less than 5% or more than 15%, it cannot be briquetted. While the residual moisture was 5%–8% or 13%–15%, the molding effect was poor, although it can be briquetted. However, when the residual moisture was 8%–13%, the briquetted effect of molding rods was good, so the ideal residual moisture for the briquette production process was 8%–13%.

**Table 2:** Effect of moisture content on raw residue briquetting.

**Moisture (%)**	<5%	5%–8%	8%–13%	13%–15%	>15%
**Can be Briquetted**	No	Almost not	Yes	Almost not	No

Dried residue with 10.0% moisture content was put into the hopper, and then the electric heating tube was opened, and the molding temperature were kept at different temperatures (50, 100, 150, 200, 250, 300, 350 and 400 °C), respectively, and the extrusion machine was started, driving the spiral propellers to push the dried residues into the molding sleeve. The briquetting effect of molding rods is shown in Table 3. When the molding temperature was less than 150 °C or more than 400 °C, it cannot be briquetted. When the molding temperature was 250–400 °C the briquetting effect is poor, and there were some cracks on the molding rods’ surface. When the molding temperature was 200 °C, the briquetting effect of the molding rods was good, there were no cracks and they were not carbonized on surface, and the molding rods output speed was the most fast of all.

**Table 3:** Effect of temperature on raw residue briquetting.

Briquetting Temperature (°C)	50	100	150	200	250	300	350	400
Can be briquetted	No	No	Yes	Yes	Yes	Yes	Almost not	No
Cracks on surface	——	——	Yes	No	Yes	Yes	A lot	——
Carbonized on surface	——	——	No	No	A little	Partial	Totally	Seriously
Briquetting rate (kg/min)	——	——	2.1	2.0	1.8	1.8	1.7	——

### 2.2. Pyrolysis Curves and Yields of Pyrolysis Products

**Figure 1:**
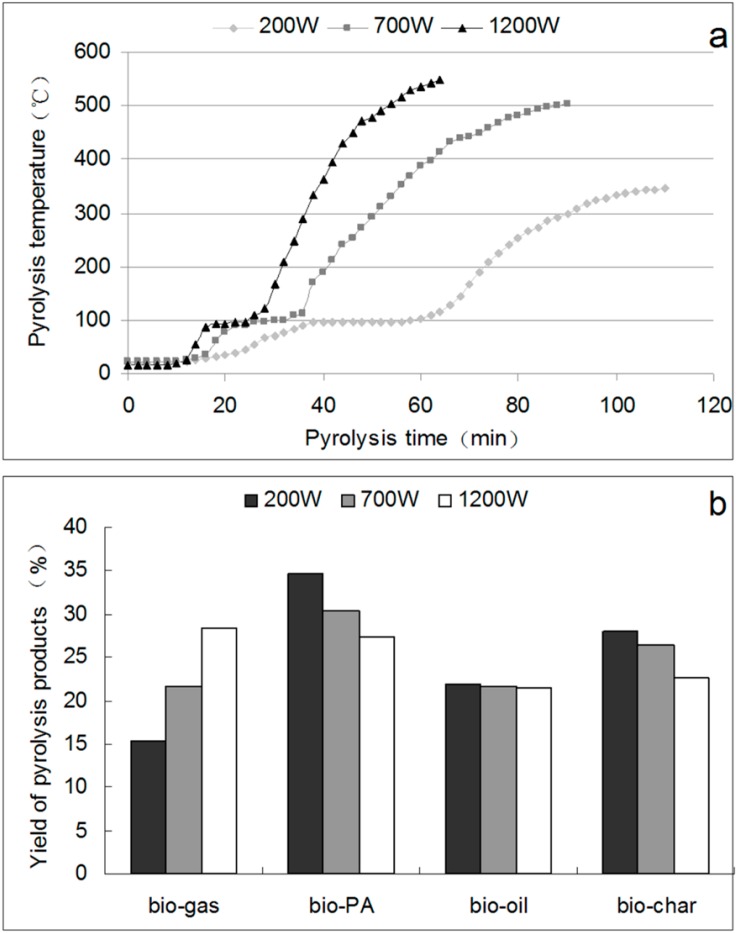
Pyrolysis curves (**a**) and the yields of pyrolysis products (**b**) generated with different heating powers.

The pyrolysis curves shows the pyrolysis temperature against pyrolysis time. The yields of the pyrolysis products, including bio-gas, bio-PA, bio-oil, and bio-char, are shown in Figure 1. The pyrolysis curves (Figure 1a) showed that with a higher heating power, the temperature increased more rapidly and the pyrolysis rate was higher.

The maximum final temperature of the pyrolysis reaction was close to 600 °C (1200 W). Depending on the thermal environment and the final temperature, pyrolysis will yield mainly biochar at low temperatures (<450 °C) when the heating rate is quite slow, and mainly gases at high temperatures (>800 °C) with rapid heating rates. At an intermediate temperature and under relatively high heating rates, the main product is bio-oil. From Figure 1b, the yields of bio-oil pyrolysis with different powers were similar, and the yields of bio-oil were 21.5% (200 W), 21.4% (700 W), and 21.4% (1200 W), respectively, of the total pyrolysis products. However, when the pyrolysis power increased, the bio-gas yield increased and the bio-PA and bio-char yields decreased, the PA yield with 200 W pyrolysis was 34.5% of the total pyrolysis products, more than that with pyrolysis at higher power 30.4% (700 W), and 27.5% (1200 W), respectively. The char yield with 200 W pyrolysis was 28.4% of the total pyrolysis products, more than that with pyrolysis at higher power 26.1% (700 W), and 22.5% (1200 W), respectively. Thus, to obtain high yields of bio-PA and bio-char, a low power pyrolysis (200 W) should be used. The mass difference from input and output products was determined by the product mass of non-condensable gas. The bio-gas yields were 15.6% (200 W), 22.1% (700 W), and 28.6% (1200 W) of the total pyrolysis products, respectively. Therefore, the higher the pyrolysis power, the faster the pyrolysis rate was, and the larger the amount of non-condensable gas produced was.

### 2.3. Separation of PA from Bio-Oil

**Figure 2:**
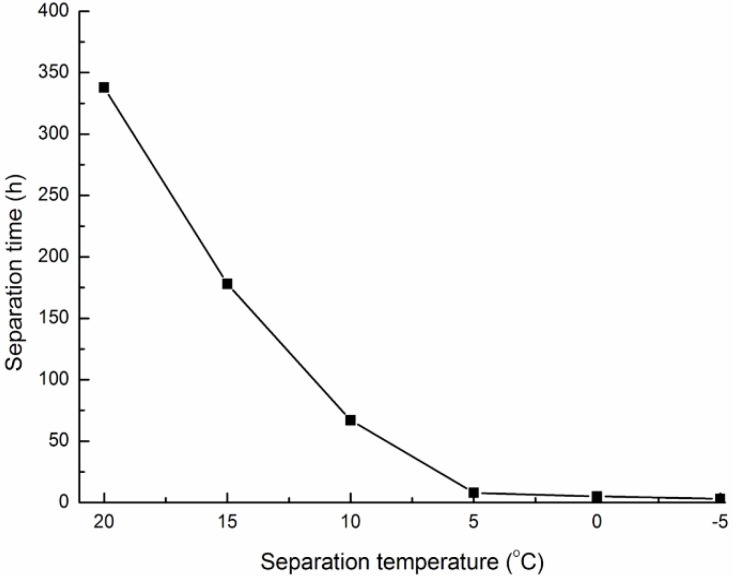
Effect of temperature on time required to separate bio-PA and bio-oil.

The condensate collected from the condenser was a mixture of PA and bio-oil, and separation by cooling was a simple way to separate the bio-PA (the upper layer) from the bio-oil (the lower layer). To investigate the effect of the separation temperature on separation time, five 200 mL aliquots of condensate were accurately measured and then poured into a separatory funnel, cooled at different temperatures (−5°C–20 °C). The volume of PA and bio-oil were recorded every hour, and the separation time was recorded at the time at which the volumes stopped changing. The separation curve for PA and bio-oil is shown in Figure 2.

Lower cooling temperatures gave better separation of the two phases than higher temperatures, and this reduced the separation time. Separation of the condensate at 20 °C required nearly 350 h for complete separation. The separation rate was higher below 5 °C, and the condensate was completely separated within 12 h. If the condensate was separated at a temperature below 0 °C, it would need refrigeration, which would increase energy consumption. Therefore, 5 °C was selected as the operation temperature to separate bio-PA from bio-oil. After stratification in the closed separatory funnel, the bio-oil (the lower layer) was removed and the antioxidant capacities of the bio-PA was determined.

### 2.4. Determination of Total Phenolics Content

The Folin-Ciocalteu assay is a fast and simple method to rapidly determine the content of phenolics in samples. Most of the work dealing with the content of phenolics in natural products uses gallic acid as a standard, and the content of phenolics in this work is expressed as gallic acid equivalents to facilitate comparison to earlier studies [37]. As shown in Table 4, the total phenolic content of PA-200 (pyrolysis at 200 W) was higher than that of PA-700 (pyrolysis at 700 W) and PA-1200 (pyrolysis at 1200 W), because the phenolic compounds were not destroyed (*i.e*., pyrolyzed) under the low temperature conditions. Furthermore, some of the water-soluble phenolic compounds were extracted into water under the lower temperature conditions, because of slow evaporation of moisture from the residue. Consequently, the total phenolic content of PA-200 was the highest, and PA-200 was selected for the antioxidant activity tests. As shown in Table 4, in all the PA extracts, the total phenolic contents of DMEP and MEP were higher than those of EFEP and TFEP. This means the phenolic compounds in PA were more likely to be soluble in dichloromethane and methanol.

**Table 4:** Total phenolic content of PA extracts.

Heating Power (W)	Symbols	Gallic Acid Equivalents (mg/g)			
		EFEP ^a^	DMEP ^b^	MEP ^c^	TFEP ^d^
200	PA-200	2.36 ± 0.05	3.91 ± 0.13	3.79 ± 0.14	2.14 ± 0.10
700	PA-700	2.35 ± 0.12	3.79 ± 0.11	3.47 ± 0.15	1.99 ± 0.06
1200	PA-1200	2.30 ± 0.13	2.81 ± 0.14	2.69 ± 0.10	1.58 ± 0.08

^a^ EFEP: ethyl formate extract phase of pyroligneous acid; ^b^ DMEP: dichloromethane extract phase of pyroligneous acid; ^c^ MEP: methanol extract phase of pyroligneous acid; ^d^ TFEP: tetrahydrofuran extract phase of pyroligneous acid.

### 2.5. Ferric Reducing Power

**Figure 3:**
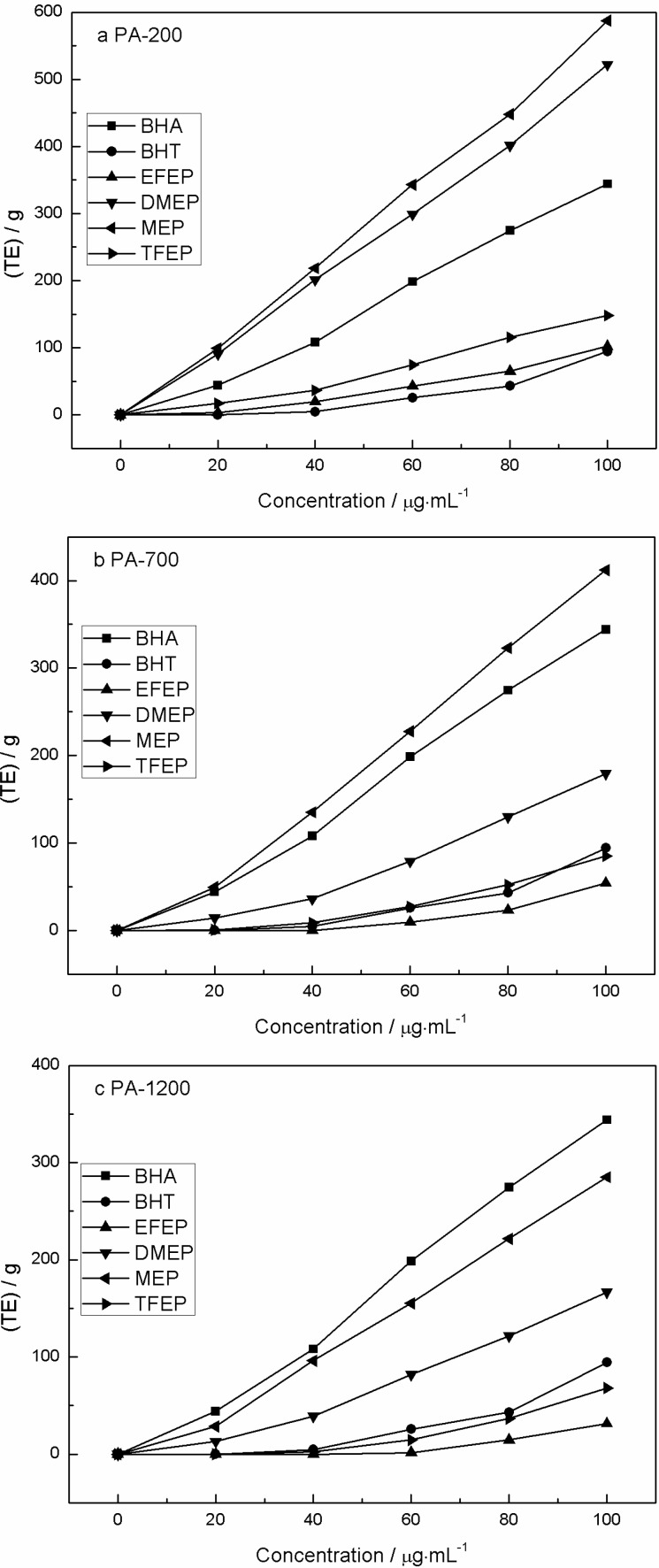
Reducing antioxidant capacities of PA extracts after (**a**) pyrolysis at 200 W; (**b**) pyrolysis at 700 W; (**c**) pyrolysis at 1200 W.

The antioxidant potentials of the PA extracts were estimated from their ability to reduce TPTZ-Fe(III) to TPTZ-Fe(II) [38]. A higher absorbance indicated a higher ferric reducing power. The reducing antioxidant power of each sample is expressed as Trolox equivalents, and the reducing antioxidant power of solvent was deducted as a blank. Figure 3 shows the reduction capacities of the various extracts from samples subjected to different heating powers during pyrolysis. Figure 3a (PA-200) shows that the reduction capacities of MEP and DMEP at 100 µg/mL were 587.55 ± 22.50 and 522.05 ± 32.20 (TE)/g, respectively. These results were higher than those of EFEP (102.35 ± 4.20 (TE)/g) and TFEP (148.05 ± 8.43 (TE)/g) at the same concentration. The reduction capacities of BHA and BHT at 100 µg/mL were 344.05 ± 11.11 (TE)/g and 94.35 ± 2.34 (TE)/g, respectively. Figure 3b (PA-700) and Figure 3c (PA-1200) show the same trends. The reducing power order of PA-200 was MEP > DMEP > BHA > TFEP > EFEP > BHT, while the order of PA-700 was MEP > BHA > DMEP > BHT > TFEP > EFEP, and the order of PA-1200 was BHA > MEP > DMEP > BHT > TFEP > EFEP.

### 2.6. DPPH Free Radical Scavenging Activity

**Figure 4:**
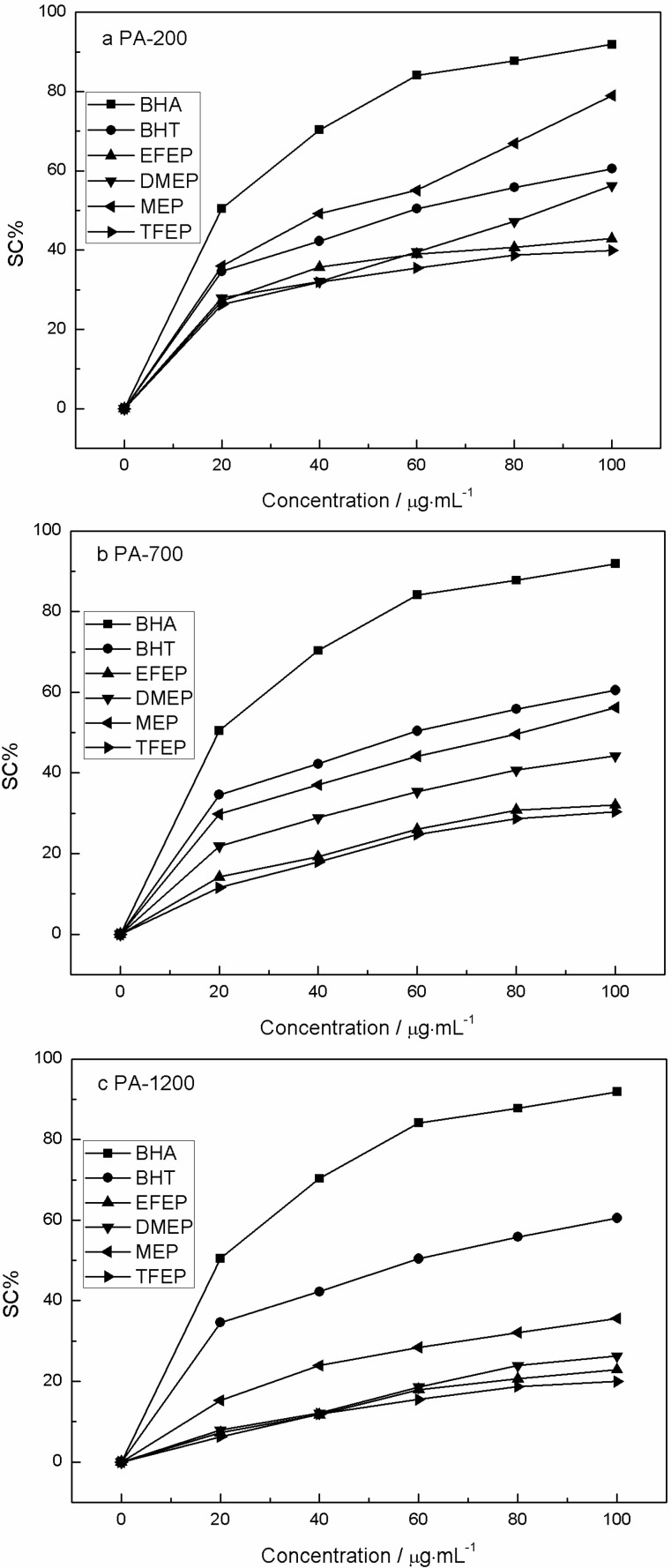
DPPH scavenging activities of PA extracts after (**a**) pyrolysis at 200 W; (**b**) pyrolysis at 700 W; (**c**) pyrolysis at 1200 W.

DPPH is a stable free radical, and can accept an electron or hydrogen free radical to reach a steady state [39]. Consequently, DPPH has been widely used to determine the free-radical scavenging abilities of various samples. When the scavenging rate of DPPH is 50%, the corresponding value of sample concentration is SC50. So, a lower SC50 value indicates the stronger antioxidant activity. As shown in Figure 4, an obvious increase in SC% value occurred when the concentration of PA increased.

Thus, the DPPH scavenging activities decreased order was MEP > DMEP > EFEP > TFEP. The DPPH scavenging activity of PA-200 (Figure 4a) was higher than that of PA-700 (Figure 4b) and PA-1200 (Figure 4c), as the lower heating power enhance the residence time of liquid pyrolysis products, increasing the content of water soluble bioactive small molecules [10]. In detail, the yields of pyrolysis products, such as the condenser and bio-char depend on the heating rate of pyrolysis. In other words, the lower heating power and the slower pyrolysis rate, the pyrolysis reaction is more completely, and the yield of the condenser liquid is higher. And vice the higher heating power and faster pyrolysis, enhance the solid pyrolysis products thus increasing bio-char formation [2]. When the pyrolysis power was 200 W, the SC50 values of MEP and DMEP were 41 ± 1.2 and 92 ± 2.3 µg/mL, respectively, and were lower than the SC50 values of EFEP and TFEP. The SC50 values of BHA and BHT were 19 ± 0.23 and 60 ± 0.30 µg/mL, respectively. The DPPH scavenging activities of PA-200 were in the order BHA > MEP > BHT > DMEP > EFEP > TFEP. While for PA-700 and PA-1200, the order was BHA > BHT > MEP > DMEP > EFEP > TFEP.

### 2.7. Chemical Composition of PA Extracts

The compositions of the *S. chinensis* PA extracts were analyzed by GC-MS. Results were accepted when a match >90% was obtained. The relative contents of the compounds were determined using the normalization method. Among the pyrolysis powers tested, 200 W pyrolysis gave the highest yield of PA, and PA-200 had the highest total phenolic content. Therefore, we used PA-200 for the antioxidant test, and extracted it with four different organic solvents. The GC-MS results from this study are shown in Table 5.

**Table 5:** Volatile compounds in PA-200 extracts.

No.	Retention Time(min)	Compounds	CAS Number	Molecular Formula	RA% ^a^			
					EFEP ^b^	DMEP ^c^	MEP ^d^	TFEP ^e^
1	5.498	Pyridine, 3,4-dimethyl-	000583-58-4	C_7_H_9_N	——	——	——	9.07
2	6.106	2,5-Furandione, 3-methyl-	000616-02-4	C_5_H_4_O_3_	16.43	7.20	17.06	14.35
3	6.489	2-Cyclopenten-1-one, 3-methyl-	002758-18-1	C_6_H_8_O	4.89	6.87	——	2.82
4	7.078	Formic acid phenyl ester	001864-94-4	C_7_H_6_O_2_	19.88	20.89	17.80	18.39
5	8.065	1,2-Cyclopentanedione, 3-methyl-	000765-70-8	C_6_H_8_O_2_	2.50	——	——	——
6	8.686	Ethylidenecyclobutane	001528-21-8	C_6_H_10_	1.70	3.15	——	1.79
7	9.261	p-Cresol	000106-44-5	C_7_H_8_O	10.41	16.24	22.84	10.35
8	9.429	Phosphonofluoridic acid, ethyl-, nonyl ester	171741-07-4	C_11_H_24_FO_2_P	9.57	12.51	9.50	8.85
9	9.755	2,4(1H,3H)-Pyrimidinedione, 5-amino-	000932-52-5	C_4_H_5_N_3_O_2_	9.98	12.61	9.29	9.07
10	9.929	4-Piperidinone, 2,2,6,6-tetramethyl-	000826-36-8	C_9_H_17_NO	2.41	3.74	1.87	2.29
11	9.996	1-Hexene, 2-methyl-	006094-02-6	C_7_H_14_	0.71	——	2.38	——
12	10.243	2,5-Pyrrolidinedione, 1-ethyl-	002314-78-5	C_6_H_9_NO_2_	1.21	——	2.59	——
13	10.294	4-Pyridinol	000626-64-2	C_5_H_5_NO	1.66	——	2.83	——
14	10.510	Succinimide	000123-56-8	C_4_H_5_NO_2_	2.03	7.71	——	2.87
15	10.698	Glutarimide	001121-89-7	C_5_H_7_NO_2_	3.60	——	6.04	3.73
16	10.869	4,5,6-Pyrimidinetriamine	000118-70-7	C_4_H_7_N_5_	2.23	2.65	——	3.68
17	10.926	3,3-Dimethylpyrrolidine-2,5-dione	003437-29-4	C_6_H_9_NO_2_	1.56	——	——	3.27
18	11.261	Creosol	000093-51-6	C_8_H_10_O_2_	1.24	——	——	1.95
19	11.490	3-Pyridinol, 2,6-dimethyl-	001122-43-6	C_7_H_9_NO	——	——	——	0.54
20	11.655	1,4,3,6-Dianhydro-alpha-d-glucopyranose	100009-81-8	C_6_H_8_O_4_	5.01	3.51	——	2.09
21	13.578	Hydroquinone	000123-31-9	C_6_H_6_O_2_	——	——	5.75	——
22 ^f^	33.801	Phenol,2,2'-methylenebis[6-(1,1-dimethylethyl)-4-methyl-	000119-47-1	C_23_H_32_O_2_	2.98	2.92	2.05	4.89

^a^ RA: relative area of total peak area (removed the blank solvent); ^b^ EFEP: ethyl formate extract phase of pyroligneous acid; ^c^ DMEP: dichloromethane extract phase of pyroligneous acid; ^d^ MEP: methanol extract phase of pyroligneous acid; ^e^ TFEP: tetrahydrofuran extract phase of pyroligneous acid; ^f^ Compound No. 22: Butylated hydroxytoluene (BHT) is added in the solvent to keep the stability of solvent, is not the composition of PA.

**Figure 5:**
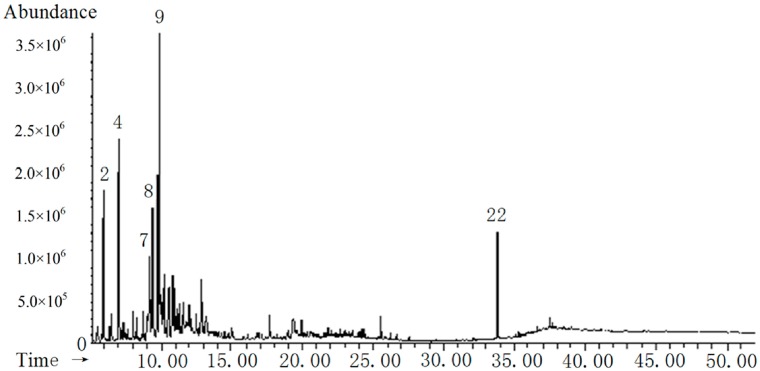

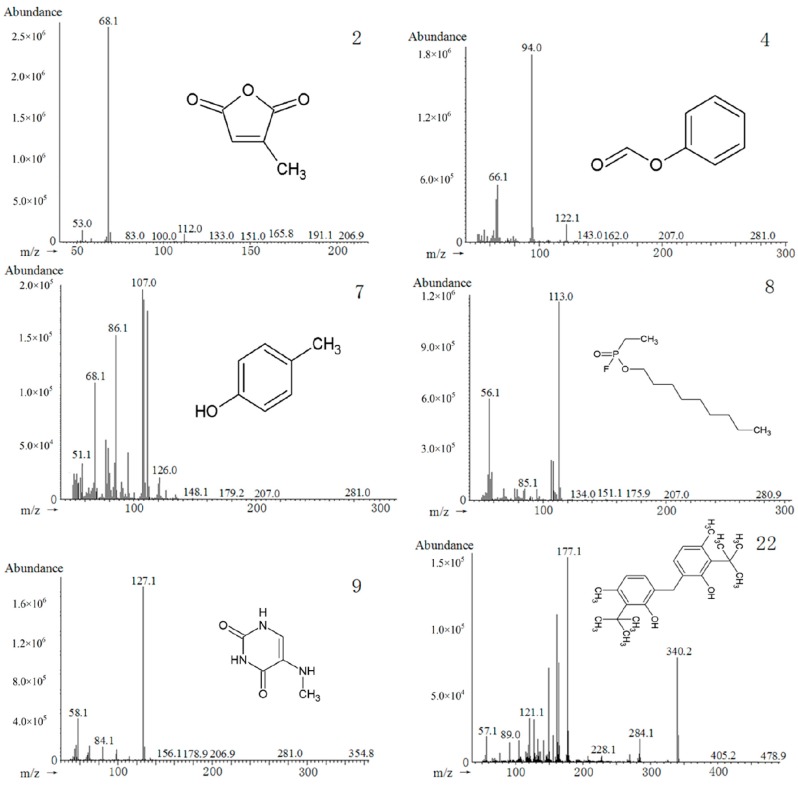
Total ion chromatogram and the main constituents of PA-200 MEP.

According to previous studies, the thermal degradation characteristics of wood materials or plants are strongly influenced by their chemical composition (cellulose, hemicellulose, and lignin) [40,41,42]. Biomass undergoing pyrolytic reactions at high temperatures forms different compounds. Saccharides, including cellulose, hemicellulose and pectin, are thermally degraded into ketones, alcohols, and furan and pyran derivatives. However, lignin is converted into phenol, guaiacol, syringol, pyrocatechol, and their derivatives, which are dissolved in the bio-oil layer [39]. After cryogenic separation, smaller molecules partition into the water layer (*i.e*., the PA layer) and larger molecules in the bio-oil layer. The gas chromatograms and the main constituents of MEP are shown in Figure 5.

The compound numbers in Figure 5 correspond to those in Table 5. The main constituents of MEP were *p*-cresol (22.84%), formic acid phenyl ester (17.80%), and furandione (17.06%). The main constituents of DMEP were *p*-cresol (16.24%), formic acid phenyl ester (20.89%), and furandione (7.20%). However, which in EFEP and TFEP was slightly lower, and the antioxidant activity of MEP and DMEP was higher than that of EFEP and TFEP. Therefore, in combination with the results of total phenolics content, ferric reducing power, and free radical scavenging activity, the phenolic hydroxyl and polyenes played an important role in improving the oxidative resistance of PA after pyrolysis. Twelve compounds from DMEP and MEP were identified, and for EFEP and TFEP, 19 and 17 compounds were identified, respectively. BHT (No. 22 compounds), which was added to the solvent to maintain its stability, was present in small amounts (<5.0%).

These results indicate that compounds containing the phenolic hydroxyl group are functional components responsible for the reduction of oxidants and scavenging of free radicals. This conclusion is supported by similar results obtained in other studies [15,39].

## 3. Experimental Section

### 3.1. Material

#### 3.1.1. Chemical Reagents

Folin-Ciocalteu’s reagent, 1,1-diphenyl-1-picrylhydrazyl (DPPH, 95%), gallic acid, 2,4,6-tripyridyl-S-trizine (TPTZ), 6-hydroxy-2,5,7,8-tetramethylchromane-2-carboxylic acid (Trolox), butylated hydroxyanisole and butylated hydroxytoluene (BHT) were purchased from Sigma-Aldrich (St. Louis, MO, USA). All other chemicals of analytical grade and were obtained from Sinopharm Chemical Reagent Co., Ltd (Beijing, China). Reverse osmosis Milli-Q water (Millipore, Billerica, MA, USA) was used for all solutions and dilutions.

#### 3.1.2. Raw Materials

*S. chinensis* fruit were purchased from San Keshu Trading (Heilongjiang, China) and identified by Professor Shao-quan Nie from the Key Laboratory of Forest Plant Ecology, Northeast Forestry University (Harbin, China). *S. chinensis* fruit were crushed and refluxed twice with an ethanol-water (80:20, v/v) solution at 90 °C for 2 h to obtain the active compounds, such as polysaccharides, anthocyanins, terpenoids, organic acids, vitamins, tannins, biphenyl cyclooctene lignans and derivatives, *etc.* And then the extracts were concentrated under the vacuum conditions, and the residues were stored under −4°C until used for thermochemical conversion.

### 3.2. Methods

#### 3.2.1. Molding Method

**Figure 6:**
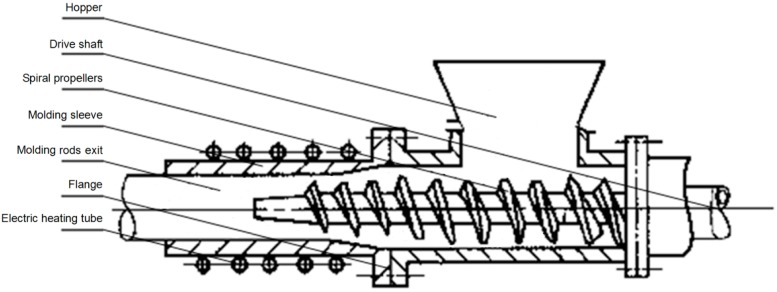
Schematic diagram of the briquetting equipment.

The dried residues prepared in Section 3.1.2 were dried and then compact molding in briquetting equipment. In the process of compact molding, there is continuous feeding and continuous discharge mode with the improved briquetting equipment. The dried residual were first put into the hopper, and then the electric heating tube and the extrusion machine were started, driving the spiral propellers put the dried residual into the molding sleeve. Finally, the molding rods were export from the exit of molding sleeve. The schematic diagram of briquetting equipment was shown in Figure 6.

#### 3.2.2. Pyrolysis Method

**Figure 7:**
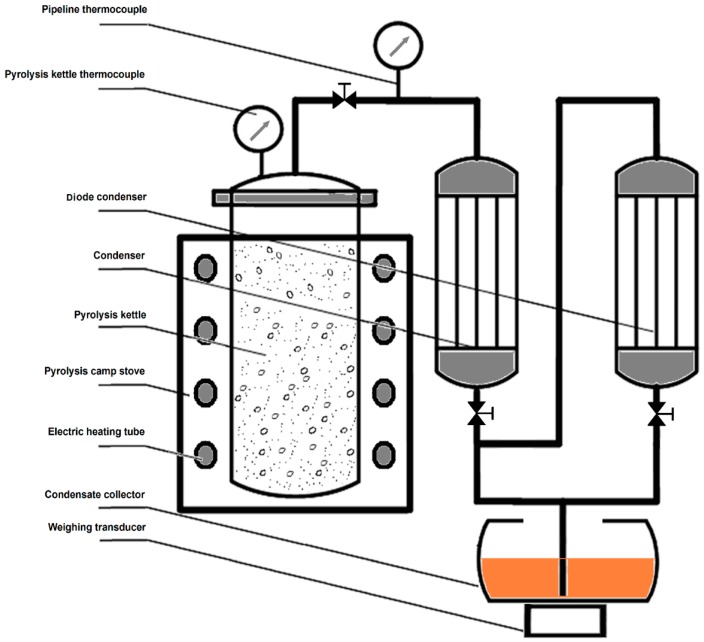
Schematic diagram of the pyrolysis equipment.

The molding rods prepared in Section 3.2.1 was heated for pyrolysis in a system consisting of a pyrolysis kettle and condensers (Figure 7). The molding rods were placed in the pyrolysis kettle, and the liquefier and pyrolysis kettle were started. The pyrolysis rate was controlled by adjusting the heating power, and the condensate was collected and the residual tar was purified. Then, the electric heating tube was opened. The end point of the pyrolytic reaction was determined by weighing the transducer. At the end point, the final weight increase of the condensate was less than 2.0%, and the valve was turned off. Condensate collected from the condenser contained PA in the upper layer and bio-oil in the lower layer. The raw PA obtained had a clear reddish-brown color similar to black tea. The solid residue remaining in the pyrolysis kettle was biochar. According to the law of conservation of mass, the mass difference from input and output products was determined by the product mass of non-condensable gas.

#### 3.2.3. Preparation Method of PA Extracts

Three PA pyrolysis products were obtained, including PA-200 (heating power 200 W, highest pyrolysis temperature 310 °C), PA-700 (heating power 700 W, highest pyrolysis temperature 440 °C), and PA-1200 (heating power 1,200 W, highest pyrolysis temperature 530 °C). Each was obtained using 200 g of *S. chinensis* fruit. The ending of the pyrolysis process was that no more yellow gas was generated and no more liquid was collected. After pyrolysis, the PAs were dried over anhydrous sodium sulfate. They were then extracted at room temperature by mixing (100 rpm) for 0.5 h with each of the following solvents: ethyl formate, dichloromethane, methanol, and tetrahydrofuran. The sodium sulfate was removed by filtration, and the organic extracts were used for antioxidant tests. While these solvents were selected for this study, they are not recommended for use in food and medicine industry, due to the residues of toxicity solvent. Especially formate, dichloromethane, and tetrahydrofuran, bacause the large consumption of organic solvent was not conducive to environmental protection. The volume of each organic solvent used was half the volume of PA. The ethyl formate extract phase (EFEP), dichloromethane extract phase (DMEP), methanol extract phase (MEP) and tetrahydrofuran extract phase (TFEP) were thick brown liquids and were stored at 4–8 °C in the dark.

#### 3.2.4. Folin Ciocalteu Assay

A colorimetric assay based on a published procedure with slight modifications [10,43] was used for estimating the total phenolic content of PA. Each sample (1 mL) was pipetted into a tube, and 50% Folin Ciocalteu’s reagent (1 mL) and 10% sodium carbonate solution (1 mL) were added. The solutions were vortex mixed for 30 s, and then left to stand at room temperature for 2 h. Absorbance measurements were recorded at 765 nm, using gallic acid to construct calibration curves. The results are reported as mean values expressed as milligrams of gallic acid equivalents per gram of sample.

#### 3.2.5. Ferric Reducing Antioxidant Power (FRAP)

The reducing antioxidant capacity of PA and its extracts were examined using a modified FRAP assay [4]. The FRAP reagent was prepared from 300 mmol/L acetate buffer (pH 3.6), 20 mmol/L ferric chloride, and 10 mmol/L 2,4,6-tripyridyl-s-triazine diluted in 40 mmol/L hydrochloric acid. The three solutions were mixed in a ratio of 25:2.5:2.5 (v/v/v). The FRAP assay was performed using reagents preheated to 38 °C. Before analysis, the initial absorbance of 3 mL of the reagent and 3 mL of acetate buffer used as a blank were measured at 593 nm. The samples (100 μL) were transferred into test tubes containing the reagent. The mixtures were shaken thoroughly and he absorbance values at 593 nm were recorded after 90 min. A higher absorbance indicated a higher ferric reducing power. The reducing antioxidant power of the sample is expressed as Trolox equivalent (TE/g), which is the ratio between the slope of the sample’s regression line and that of Trolox.

#### 3.2.6. DPPH Free Radical Scavenging Activity

The DPPH radical scavenging activities of PA and its extracts were examined using the method of Lee *et al.* [44] and compared with those of the synthetic antioxidants BHA and BHT. Briefly, a 1.0 mL sample was mixed with 2.0 mL of a methanolic solution of DPPH (35 mg/L) in a lightproof container. The mixture was shaken (100 rpm) and allowed to stand at room temperature for 30 min. Then, the absorbance was measured at 517 nm (UV-2550 spectrophotometer, Shimadzu, Kyoto, Japan) against methanol as a blank. Lower absorbance values for the reaction mixture indicated higher free radical scavenging activity. The percentage of DPPH discoloration of the samples was calculated according to the formula:

SC % = (A_0_ − A)/A_0_ × 100

where A_0_ is the absorbance of the control reaction, which contained all reagents except the test compound; and A is the absorbance of the sample. The sample concentration providing 50% inhibition (SC50) was calculated from a graph of inhibition percentage against sample concentration.

#### 3.2.7. GC-MS Analysis

GC-MS analysis was carried out on an Agilent 7890A GC system (Agilent Technologies, Palo Alto, CA, USA) fitted with a DB-17MS capillary column (30 mm × 0.25 mm, film thickness 0.25 μm) and equipped with an Agilent 7693 auto sampler, and 5975C inert XL EI/CI mass selective detector with triple-axis detector. A sample volume of 2 μL was injected manually in splitless mode. The carrier gas was helium (He) at a flow rate of 1.0 mL/min. The oven temperature was set at 60 °C for the initial 5 min, then increased to 120 °C at a rate of 10 °C/min and held for 5 min, increased to 200 °C at a rate of 10 °C/min and held for 5 min, and then increased to 280 °C at a rate of 10 °C/min and held for 15 min. The injector and detector temperatures were 280 °C and 230 °C, respectively. The pressure and flow rate of the injector were 15.0 psi and 25.0 mL/min, respectively. The MS was operated at 70 eV, and the scan range (TIC) was 50–500 *m/z*.

## 4. Conclusions

The pyrolysis process and products of *S. chinensis* fruit were studied. The yield of PA was higher with a lower pyrolysis heating power than with a higher pyrolysis heating power. Based on the total phenolic contents of the PA extracts, PA-200 was selected to test for antioxidant activities, including free radical scavenging activity and ferric reducing power. MEP and DMEP of PA-200 showed superior characteristics compared to the other extracts. Phenols and polyenes were the major components in MEP and DMEP, followed by ketones and furan derivatives. The results indicated that phenols were the functional components from *S. chinensis* fruit responsible for the reduction of oxidants and scavenging of free radicals in PA. The organic solvents in the PA extracts were vacuum evaporated, and then dissolved in ethanol, used in food, medicine, cosmetics and pesticides industry, respectively. Moreover, the further analysis of PA extracts, such as toxicity tests are necessary.
